# Automated Detection of Portal Fields and Central Veins in Whole-Slide Images of Liver Tissue

**DOI:** 10.1016/j.jpi.2022.100001

**Published:** 2022-01-20

**Authors:** Daniel Budelmann, Hendrik Laue, Nick Weiss, Uta Dahmen, Lorenza A. D’Alessandro, Ina Biermayer, Ursula Klingmüller, Ahmed Ghallab, Reham Hassan, Brigitte Begher-Tibbe, Jan G. Hengstler, Lars Ole Schwen

**Affiliations:** a Fraunhofer MEVIS, Lübeck, Germany; b Fraunhofer MEVIS, Bremen, Germany; c Experimental Transplantation Surgery, Department of General, Visceral and Vascular Surgery, University Hospital Jena, Jena, Germany; d Deutsches Krebsforschungszentrum, Systems Biology of Signal Transduction, Heidelberg, Germany; e Leibniz Research Centre for Working Environment and Human Factors at the Technical University Dortmund, Dortmund, Germany; f Department of Forensic Medicine and Toxicology, Faculty of Veterinary Medicine, South Valley University, Qena, Egypt

**Keywords:** liver, portal field, central vein, object detection, convolutional neural network, zonated quantification, CNN, convolutional neural network, CV, central vein, H&E, hematoxylin and eosin, GS, glutamine synthetase, PBS, phosphate buffered saline, PF, portal field, WSI, whole-slide image

## Abstract

Many physiological processes and pathological phenomena in the liver tissue are spatially heterogeneous. At a local scale, biomarkers can be quantified along the axis of the blood flow, from portal fields (PFs) to central veins (CVs), i.e., in zonated form. This requires detecting PFs and CVs. However, manually annotating these structures in multiple whole-slide images is a tedious task. We describe and evaluate a fully automated method, based on a convolutional neural network, for simultaneously detecting PFs and CVs in a single stained section. Trained on scans of hematoxylin and eosin-stained liver tissue, the detector performed well with an F1 score of 0.81 compared to annotation by a human expert. It does, however, not generalize well to previously unseen scans of steatotic liver tissue with an F1 score of 0.59. Automated PF and CV detection eliminates the bottleneck of manual annotation for subsequent automated analyses, as illustrated by two proof-of-concept applications: We computed lobulus sizes based on the detected PF and CV positions, where results agreed with published lobulus sizes. Moreover, we demonstrate the feasibility of zonated quantification of biomarkers detected in different stainings based on lobuli and zones obtained from the detected PF and CV positions. A negative control (hematoxylin and eosin) showed the expected homogeneity, a positive control (glutamine synthetase) was quantified to be strictly pericentral, and a plausible zonation for a heterogeneous F4/80 staining was obtained. Automated detection of PFs and CVs is one building block for automatically quantifying physiologically relevant heterogeneity of liver tissue biomarkers. Perspectively, a more robust and automated assessment of zonation from whole-slide images will be valuable for parameterizing spatially resolved models of liver metabolism and to provide diagnostic information.

## Background

### Motivation

Many hepatic processes are spatially heterogeneous on two major spatial scales: lobes and lobuli.^1^ Lobes are macroscopically distinct parts of livers.^1^ At this scale, heterogeneity is mainly due to disease states such as fibrosis,^2^^,^^3^ cirrhosis,^4^ steatosis,^5^^,^^6^ and carcinoma.^7^^,^^8^ At the tissue scale, blood flows from the portal fields (PFs) to the central veins (CVs) through capillaries (sinusoids). Tissue regions drained by the same CV are referred to as lobuli; conversely, regions supplied by the same PF are referred to as acini.^1^ Lobuli have a radius of a few 100 μm or about 12 hepatocytes in mice^9^ and 19 in humans.^1^ Heterogeneity of biological properties of the cells along the PF–CV axis is denoted as zonation.^10^, ^11^, ^12^, ^13^, ^14^ Physiological metabolic processes exhibit zonation mainly due to zonated gene and protein expression of the hepatocytes,^15^, ^16^, ^17^, ^18^ and gradients of compounds in the blood being metabolized along the PF–CV axis. Additionally, pathological alterations of hepatic tissue can be zonated, e.g., steatosis^13^^,^^19^, ^20^, ^21^ or toxic damage, as after administration of carbon tetrachloride.^22^^,^^23^

Expression of tissue biomarkers are usually assessed after visualization using histological staining techniques. Heterogeneity of biomarker expression and zonation can be quantified in digital images of histological sections by computing statistical measures of variance. For this purpose, the quantity of interest is often assessed in uniform squares^24^ or hexagonal tiles.^25^ Such uniform tilings, however, do not represent physiologically meaningful structures. A heterogeneity assessment describing physiologically relevant regions requires a tiling matching the physiological geometry instead. Such a tiling needs to be based on the respective physiological structures, i.e., locations of PFs and CVs for a lobulus-based analysis. Besides merely characterizing heterogeneity, it can be valuable to quantify tissue biomarkers of interest in physiologically relevant regions. Such quantitative parameters are particularly interesting for parameterizing models of hepatic metabolic processes involving the sinusoidal or lobular scale.^23^^,^^26^, ^27^, ^28^, ^29^, ^30^, ^31^

Tissue structures of interest can be annotated manually. This is feasible for small to moderate numbers of lobuli, but is a tedious task for large-scale analyses of multiple whole-slide images (WSIs). The previous study^21^ analyzing 34 scans required manually annotating more than 24,000 PF and CV positions. To our knowledge, automated PF and CV detection in histological WSIs of liver tissue has not yet been reported.

In the present study, serial sections of mouse liver tissue slides stained with hematoxylin and eosin (H&E) and glutamine synthetase (GS) were prepared, scanned, aligned by image registration, and manually annotated based on the H&E and GS images. It is possible to simplify the manual annotation or potentially automate it by using additional stainings such as E-Cadherin.^32^ This would, however, increase the effort for creating, staining, and scanning additional histological slides. Moreover, image registration of multiple WSIs would be necessary, including a sufficiently accurate compensation for deformations to allow robustly detecting PFs and CVs. Instead, our goal in this study was to evaluate how well automated detection of PFs and CVs works based on WSIs of single H&E-stained liver sections. Zonated quantification then still requires registering the H&E image with the scan of the staining of interest. Hence, the approach could perspectively be extended to detect PFs and CVs directly in a variety of stainings of interest.

### Related Work

Detecting PFs and CVs in WSIs of histological sections is one application of object detection in images, a computer vision task which has been investigated for decades.^33^ Various classical techniques for different applications have been proposed^34^: approaches based on template matching, geometric/context knowledge, object-based image analysis, and machine learning using previously extracted image features. These techniques work well if image features allow to reliably and robustly identify the structures of interest. However, the appearance of PFs and CVs in the histological images is highly variable, which makes defining suitable image features infeasible.

Deep learning techniques using convolutional neural networks (CNNs) are known as useful tools for object detection in generic images. This approach does not require determining hand-crafted image features to distinguish the objects of interest, but builds implicit representations of suitable image features for the CNN. Object detection and classification using CNNs can generally be implemented by two- or one-stage approaches,^35^ i.e., first detecting objects and subsequently classifying them, or doing both in a single step. Examples for two-stage approaches are the region-based CNN (R-CNN) approach^36^, ^37^, ^38^, ^39^, ^40^ and feature pyramid networks using a multiscale approach.^41^^,^^42^ One-stage approaches include variants of YOLO (You Only Look Once),^43^, ^44^, ^45^ SSD (Single-Shot multibox Detection),^46^ RefineDet,^47^ EfficientDet,^48^ and RetinaNet.^49^ The networks are often pre-trained on ImageNet,^50^ an image database comprising a vast amount of natural images. Object detection approaches are commonly compared using the COCO (Common Objects in COntext) detection datasets^51^ as a benchmark task. Most of the approaches above led the ranking of the COCO benchmark at some point.

Apart from object detection, CNNs have also been used successfully for a variety of image analysis tasks in computational pathology.^52^, ^53^, ^54^, ^55^, ^56^ Applications include classification of tissue for assessing cancer,^57^, ^58^, ^59^, ^60^ segmentation of structures of interest,^61^^,^^62^ and detection of cells ^63^^,^^64^ or nuclei.^65^ This makes CNN-based deep learning a promising approach also for PF and CV detection in histological WSIs of liver tissue.

### Contribution

The goal of this study was to develop and evaluate an automatic method for detecting PFs and CVs in WSIs of single H&E-stained mouse liver sections. We present a fully automated CNN-based method for detecting and classifying PFs and CVs in these images. We quantitatively evaluated the quality of this detection approach by comparing algorithmically determined positions to manual annotations on independent test data. Moreover, we assessed how well this detector generalizes to scans of steatotic liver tissue, a condition not present in the training data.

Two proof-of-concept applications are included as examples of how subsequent automatic analyses can build upon automated PF and CV detection: We complemented the quantitative evaluation of detection quality by a lobulus size analysis based on annotated and algorithmically detected PF and CV positions. Moreover, we quantified the zonation of selected stainings (including a negative and positive control) based on the algorithmically detected positions as a second prototypical application.

## Material and Methods

### Data Acquisition

For this study, we used three different datasets, partially from previous studies. These datasets serve different purposes: training and evaluating the PF/CV detector on mouse liver tissue (dataset A), evaluating how well the detector generalizes to steatotic mouse liver tissue (dataset B), and illustrating the application of the detector for a prototypical zonated quantification (dataset C). Dataset A consists of a combination of normal and regenerating liver tissue one and two days after partial hepatectomy. This combination was chosen to include moderate variability in the tissue appearance. Datasets A and B consist of scans of H&E- and GS-stained liver tissue. Here, the H&E images were used to train and/or evaluate the PF/CV detector, whereas the CV-specific GS staining was used to facilitate manual annotation of CVs and distinguishing them from PFs. Dataset C consists of scans of H&E-, GS-, and F4/80-stained tissue. Here, H&E staining was used to detect PFs and CVs as well as a negative control without zonated signals for the prototypical zonated quantification. The GS staining was used as a positive control, because the GS staining (signal) is known to be strictly zonated to the CV.^66^ The F4/80 staining was chosen as a prototypical application because it exhibited a visually zonated heterogeneous pattern. We deliberately chose this biologically nonspecific staining pattern rather than a biologically more relevant staining to underline the proof-of-concept nature of the zonated quantification presented here.

### Slide Preparation and Image Acquisition

#### Dataset A

Livers from C57/Bl6N mice (Charles River, Sulzfeld, Germany) were explanted before, 24 hr after, and 48 hr after 70% liver resection following the technique described in previous work.^67^ All procedures and housing of the animals were strictly carried out according to the German animal welfare legislation (reference numbers 02-123-10, 02-123-10, 02-122-12). After formalin fixation and paraffin embedding the whole livers were subjected to serial sectioning (4 μm) followed by repeated serial staining. Every 25 sections, 2 serial sections were stained with H&E and GS. Sections were scanned using a Hamamatsu slide scanner at 400-fold magnification resulting in a resolution of 227 nm per pixel. From the total of 92 H&E and GS pairs, we randomly chose a subset of 30 pairs of neighboring H&E and GS for further processing.

#### Dataset B

As a second dataset, we used scans of 35 H&E-stained slides spaced across a serial section of an entire steatotic mouse liver from a previous study.^21^ In summary, steatosis was induced in a male C57/BL6N mouse (Charles River, Sulzfeld, Germany) by feeding a methionine/choline-deficient high fat diet (E15652-94 EF R/M, high fat MCD mod. low methionine and choline experimental diet; ssniff, Sulzfeld, Germany) for four weeks. All procedures and housing of the animals were strictly carried out according to the German animal welfare legislation (reference number 02-122-12). At the end of the observation period, the animal was sacrificed and the liver explanted, fixed in 5% buffered formalin followed by paraffin embedding. Subsequently, 3 μm sections were prepared using a rotary microtome (Microm HM355S; Thermo Fisher Scientific Microm International GmbH, Walldorf, Germany). A total of 2037 slides were produced and stained in batches of 25 slides. Each batch contained, among other stainings, two subsequent slides stained with H&E and GS, respectively. The slides were digitalized using a whole-slide scanner (NanoZoomer HT 2.0, Hamamatsu Photonics K.K., Hamamatsu City, Japan; at 400-fold optical magnification) at an in-plane image resolution of 227 nm. From these images, 35 undamaged pairs of neighboring H&E and GS WSIs covering the entire liver were selected.

#### Dataset C

Mice of C57BL/6N (Charles River, Sulzfeld, Germany) were housed at the DKFZ animal facility under a constant light/dark cycle, maintained on a standard mouse diet (KLIBA NAFAG 3437) and allowed ad libitum access to water and food. All animal experiments were approved by the governmental review committee on animal care of the state Baden Württemberg, Germany (reference number G33-17). For liver extraction, anesthesia was carried out by intraperitoneal injection of 11.25 mg ketamine hydrochloride 10 % (w/v) (Bayer Health Care, Leverkusen, Germany) per 100 mg body weight, 1.65 mg xylazine hydrochloride 2 % (w/v) (Pfizer, Berlin, Germany) per 100 mg body weight and 1 ml acepromazine per 100 mg body weight.

After anesthesia, the mouse thorax was opened and the liver taken out. The left lobe was fixed in 4 % paraformaldehyde (PFA) (Roti-Histofix, Roth P 087-5, Carl Roth, Karlsruhe, Germany) at 4°C for 2 days and 1–1.5 cm were used for paraffin sections. Subsequently, PFA was replaced by phosphate-buffered saline (PBS) and tissue was embedded in paraffin.

Immunohistochemistry and H&E staining were performed as previously described.^68^, ^69^, ^70^, ^71^ Briefly, formalin-fixed paraffin-embedded liver tissue sections of 5 μm thickness were used. Following de-paraffinization and rehydration steps, antigen retrieval was performed by boiling the tissues in citrate buffer, pH 6.0. Subsequently, endogenous peroxidases were blocked by immersing the tissues in 0.3% hydrogen peroxide in methanol for 10 min. For further blocking of nonspecific bindings, the tissue sections were incubated in 1% bovine serum albumin in PBS for 2 hr. Subsequently, the tissues were incubated with primary antibodies against glutamine synthetase (GS; 1:1000; BD Biosciences, Heidelberg, Germany, Cat. number: 610517) and F4/80 (1:50; AbD Serotec, Hercules, CA; Cat. number: MCA497) overnight in a humid chamber at 4°C. Following washing steps, the tissue sections were incubated with appropriate horseradish peroxidase-conjugated secondary antibodies: anti-mouse Immunglobulin G peroxidase (IgG-pod) (1:500; Sigma-Aldrich, St. Louis, MI; Cat. number: A3682), and rabbit anti-Rat IgG (H+L) (1:1000; Linaris GmbH, Dossenheim, Germany; Cat. number: BA-4001) for 2 hr in a humid chamber at room temperature. Subsequently, antibody bindings were visualized by staining with 3,3’-diaminobenzidine solution (Vector Laboratories, UK) for 2–5 min, depending on color development, and counterstained with Mayer’s hematoxylin in order to visualize nuclei. Finally, the stained sections were dehydrated, by passing in an ascending ethanol series, and preserved by mounting with entellan. Whole-slide scanning was performed using an Axio Scan.Z1 slide scanner (Zeiss, Jena, Germany) as previously described.^72^ The stained slides were scanned at 221 nm per pixel.

### Data Preparation and Image Annotation

To accelerate further processing, the full-resolution WSIs were resampled to a lower resolution by a factor of 4 in each direction. Images of neighboring slides with H&E and GS stainings in dataset A were registered by subsequent pre-alignment, affine transformation, and non-linear, intensity-based registration.^73^ The H&E images of dataset A were manually annotated by an expert with axis-aligned rectangles (named ‘tight boxes’ hereafter) around the PFs and CVs. This task was facilitated using a software allowing to switch between viewing the H&E image and the transformed GS image. Annotations of PFs and CVs for the H&E images in dataset B were available from the previous study^21^ as points. Neighboring WSIs with H&E, GS, and F4/80 stainings in dataset C were registered as described above. An overview of which dataset (with how many slides/annotations) was used for which part of this study is given in Table 1.

**Table 1:** Overview of datasets and which part of the study they were used for (calibrating and evaluating detector, proof-of-concept applications).

Dataset, subset	Slides	Annotations	Usage
A, training	22	8369	Calibrating detector
A, validation	4	2097	Calibrating detector
A, test	4	1567	Evaluating detector, lobulus size computation
B	35	46623	Evaluating detector on out-of-distribution data
C	3	n/a	Zonated quantification

### Portal Field and Central Vein Detection on Whole-Slide Images

**Figure 1:**
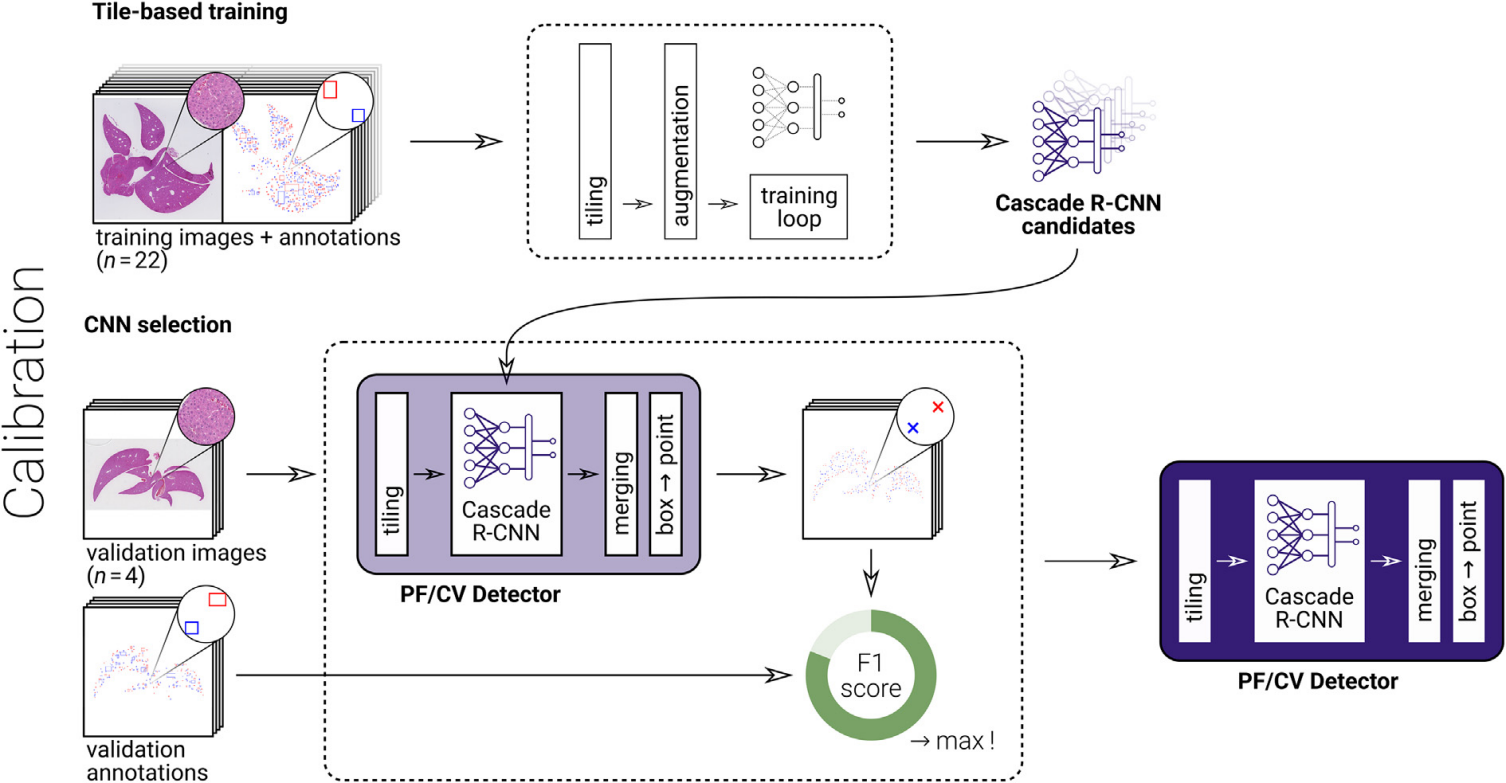
Illustration of the calibration of the portal field and central vein detector: Manual annotations on images were used to train a Cascade-R-CNN over a number of epochs. Detecting structures on whole-slide images demanded merging tile-wise information, reducing detected boxes to their midpoints. The detection on validation images with manual annotations was compared to choose the network with maximum F1 score. This allowed minimizing overfitting to the training data.

We used a tile-based approach^74^ to detect PFs and CVs on WSIs. The scans of dataset A were first divided into tiles, a CNN then detected PFs and CVs on these tiles. Finally, the detected locations were merged back into the geometry of the WSI. The detection algorithm for the entire scans was calibrated in two steps: First, a CNN for tile-based detection was trained for a fixed number of epochs. Then, the network weights performing best in the actual task of PF/CV detection on WSIs was selected using independent validation data to avoid overfitting. These steps are illustrated in Fig. 1.

We divided the 30 WSIs of dataset A into 22 training, 4 validation, and 4 test images, allowing for algorithm calibration and independent evaluation. From the training images, we extracted tiles of size *s* × *s* = {512^2^, 640^2^, 768^2^, 896^2^, 1024^2^, 1280^2^} with 50% overlap between tiles per direction, i.e., tiles are centered at *s*(1 + *i*)/2, *s*(1 + *j*)/2 for integer *i* ∈ {0, 1, 2, …} and *j* ∈ {0, 1, 2, …}. We sampled a balanced number (50:50 split) of tiles with and without PF/CV box annotations. A tile was considered ‘with box’ if there was at least one box annotation with the box center lying inside the interior [*s*/4, 3*s*/4] × [*s*/4, 3*s*/4] of the tile, otherwise the tile was considered ‘without box’ (see Supplementary Figure 1).

### Training a Convolutional Neural Network for Tile-Based Recognition

After an initial exploration of the detection quality when using different CNN architectures, Cascade R-CNN^39^ with a ResNeXt101^75^ backbone pre-trained on the ImageNet dataset^50^ looked most promising. We thus chose this architecture for manual optimization of hyperparameters. This CNN determines bounding boxes of the objects of interest and classifies them. We used smooth *L*_1_ loss for the bounding box regression and cross entropy for the classification as suggested in earlier work.^39^ The training ran for a fixed number of 24 epochs with a learning rate of 0.01 and a batch size of eight, on four GPUs in parallel. The learning rate was decreased 10-fold in epochs 16 and 22. The images were randomly flipped horizontally for moderate data augmentation. In analogy to the pre-training, every color channel of each tile is normalized for mean and standard deviation. The training was implemented using the *MMDetection* toolbox v2.0.^76^

### Merging Tile-based Detection for Assessing Whole-Slide Images

Obtaining object positions in WSIs requires merging the tile-based information. Merging involves three steps: (a) mapping the tile-based detection result in coordinates relative to the tile to the actual location relative to the WSI; (b) combining detected boxes for structures spanning multiple tiles; and (c) computing center points of the boxes. Step (a) is implemented by adding the location of the tile as an offset to the local coordinates. For step (b), we exploit that overlapping tiles are sufficiently large to cover our structures of interest and that best detection results are obtained in the middle of tiles: If boxes were contained in the interior [*s*/4, 3*s*/4] × [*s*/4, 3*s*/4] of a tile of size  *s* × *s*, they were kept. These tile interiors are mutually disjoint, thus such boxes cannot occur multiple times. Otherwise, a structure may be detected in up to four boxes. In this case, the box maximizing the area intersecting with the interior of the corresponding tile was selected. Box centers in step (c) are computed as the arithmetic mean of the vertices.

### Calibrating the Portal Field and Central Vein Detector

The merging step allowed determining points on WSIs automatically. We quantified the detection quality using standard precision, recall, and F1 score^77^ for the two classes, PF and CV. True positives were ground truth boxes for which a point inside the box of the same class was present. False positives were points outside ground truth boxes of the same class. False negatives were ground truth boxes for which no point of the same class was obtained by the algorithm. There is no meaningful number of true negatives in this detection problem, so the F1 score was chosen to quantify the performance. Detecting PFs and CVs is of equal importance, so the arithmetic mean of the two respective F1 scores was used to quantify the quality of the detection. The weights of the Cascade R-CNNs were saved after each training epoch. Among these, the one with highest F1 score for the validation data was selected.

### Evaluation of the Whole-Slide Detection

**Figure 2:**
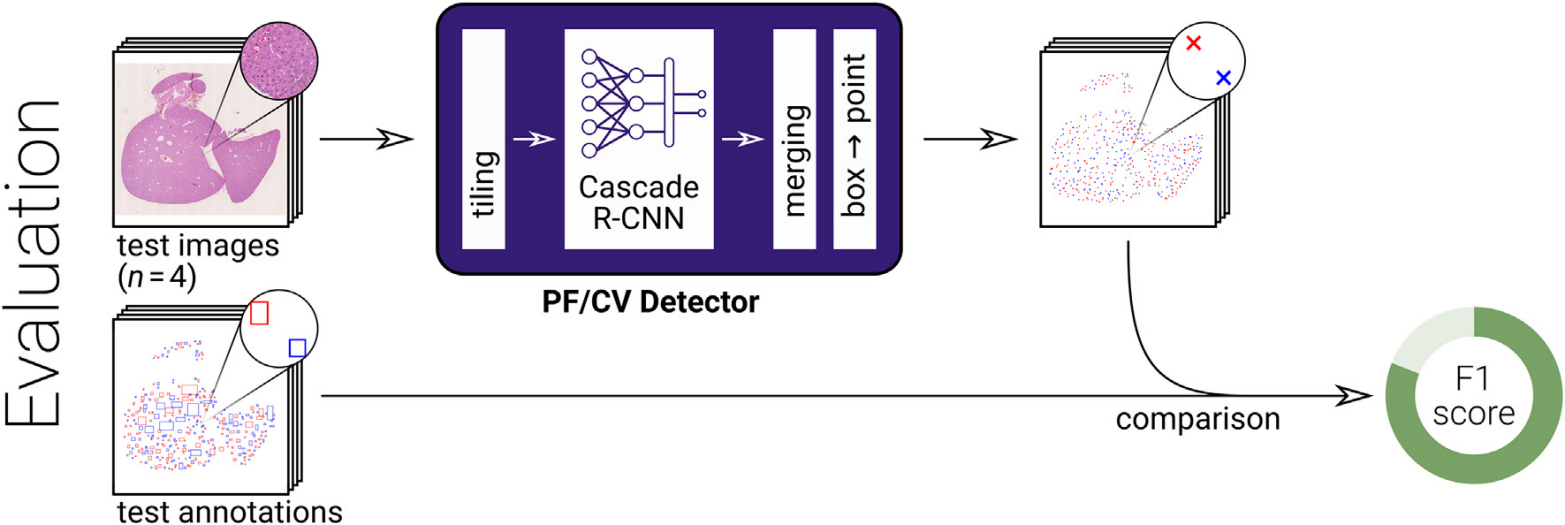
Illustration of the evaluation of the portal field and central vein detector: Algorithmically detected points for the whole-slide images were compared to manual box annotations via F1 scores.

The detection quality was evaluated on the independent test data from dataset A as performed for calibration, see Fig.  2. The evaluation was applied for all tile sizes defined above to determine the tile size for which the PF/CV detector produced best results.

In addition, the detector performance was evaluated on data with a pathological condition not present in the training data. For this purpose, the algorithmic detection was applied to H&E-stained slides of steatotic tissue from dataset B, using the optimal tile size of 1024^2^ determined in the previous step. The manual annotations were points rather than boxes, requiring the evaluation to be adapted. The box-to-midpoint conversion in the detector was omitted and the automatically determined boxes were compared to the manually annotated points in the same manner as before. This reverses the definition of ‘false positives’ and ‘false negatives’ compared to the evaluation above, but leaves ‘true positives’ unaffected. Consequently, the meaning of ‘precision’ and ‘recall’ is exchanged, but the F1 score remains unaffected and results are directly comparable.

### Proof-of-Concept Applications

**Figure 3:**
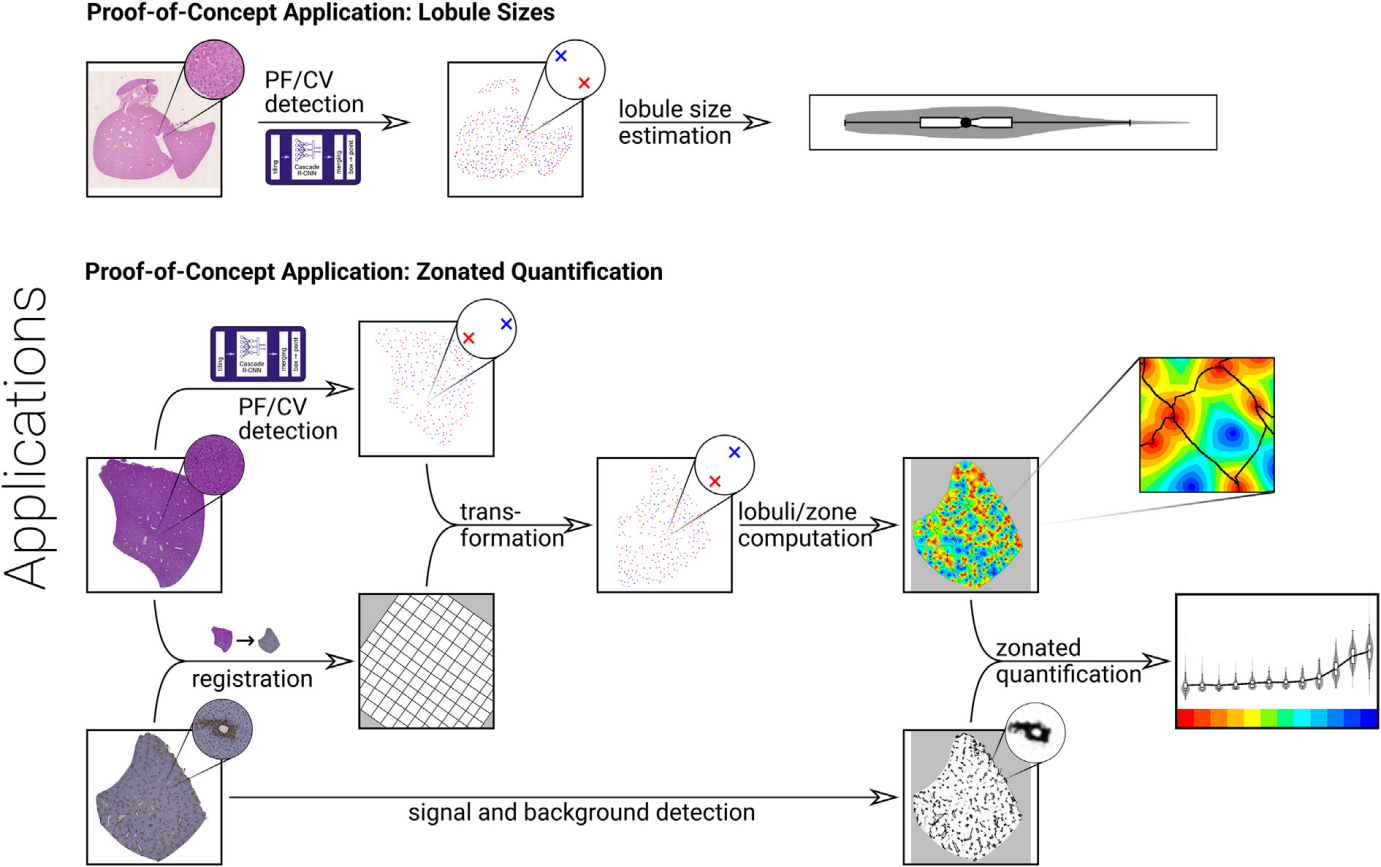
Illustration of the proof-of-concept applications using our portal field and central vein detection. Top: From the detected points, we computed the distribution of same-class nearest-neighbor distances to approximate acinus and lobulus radii, moreover, we computed lobulus areas via a watershed transform. Bottom: For consecutive sections with a staining of interest and with H&E staining, respectively, we used the detected points, transformed to the staining of interest via image registration, to compute a tiling in lobuli and zones. We then quantified the signal intensity of the staining of interest in this physiologically relevant tiling to obtain a zonated quantification.

Detecting PFs and CVs in liver tissue histological images is the basis for subsequent analyses, which we demonstrated here by two proof-of-concept applications: computation of lobulus sizes and zonated quantification, see Fig.  3.

One goal of computing lobulus sizes was to compare our present results to literature and thus check them for plausibility. Additionally, the lobulus sizes computed based on manual annotations were compared to sizes based on algorithmically obtained positions. This complements the detector performance assessment above by lobulus size as a more meaningful quantity of interest than the F1 score. However, it is not an independent validation: the same dataset is analyzed, and the lobulus size computation is based on additional assumptions.

Zonated quantification goes one step further by assessing additional image information besides anatomical geometry. Two building blocks were needed: extracting the signal to be quantified from the staining of interest, and computing zones and lobuli in which to quantify the signal.

### Determining Zones and Lobuli

We used the approach from^21^ for tessellating the non-background portion of each WSI into lobuli for performing the lobulus size analysis and for the zonated quantification. For each pixel midpoint, the ‘portality’ was computed, defined as the relative distance to the closest PF in the CV–PF direction,^21^ ranging from 0 at CVs to 1 at PFs. From the portality, catchment basins of the preflooded watershed transform^78^ were computed. For each basin, 12 zones were defined by quantizing the portality into value ranges of width 1/12. Each pixel of the whole-slide image thus belonged to one zone in one catchment basin. The result is represented as two label images with the zone and catchment basin indices, respectively.

Computed lobuli are obtained from the catchment basins: The entire image, also beyond the tissue boundary, is tessellated in basins. Masking out the background thus allows interpreting each masked basin as a computed lobulus. A background mask for this purpose was determined as follows: First, the image was converted to grayscale using the weighted average *Y* = 0.299 · *R* + 0.587 · *G* + 0.114 · *B*. Applying Otsu’s method^79^ to threshold the grayscale image by maximizing the inter-class intensity variance yielded a mask approximately distinguishing foreground and background. All enclosing contours larger than 0.785 mm^2^ (corresponding to a circle of 1 mm diameter) were identified, as they most likely reflect the main tissue. Enclosed empty spaces larger than 0.00785 mm^2^, e.g., between two tissue segments, were ignored. Finally, the edges were smoothed by a median blur and a morphological opening. These steps were implemented in Python using OpenCV,^80^ the result of masking out the background is represented as a label image containing lobulus indices.

### Computing Lobulus Sizes

Based on the CV points in the test data (subset of dataset A), radii of lobuli were approximated as half the distance from each CV point to its nearest same-class neighbor. In the same way, acinus radii based on the PF points were approximated. The computation of nearest neighbor distances was implemented using KDTrees^81^ from the Python package SciPy.^82^ Moreover, the sizes (areas) of lobuli were computed from the pixel counts and the resolution of the lobulus label images obtained as described above.

### Simple Color-Based Staining Signal Detection

We used a simple color-deconvolution-based approach to extract the relevant signals from WSIs of the stainings of interest^83^: First, the RGB image channels of the WSIs in dataset C were transformed to an optical density image. Then, its color values were projected onto the two principle colors, which were detected automatically by the Macenko method^84^ (H&E, GS) or estimated manually in the image (F4/80). The Python package Staintools^85^ was used to perform the signal decomposition. It resulted in two separate signal intensity images corresponding to the principal colors for all stainings. These intensity images separate the hematoxylin and eosin channels for the H&E images, whereas only one channel is relevant in the GS and F4/80 images, respectively. The channel decomposition is illustrated in Supplementary Figs. 2, 3, and 4.

### Zonated Quantification

Zonated quantification combines the two quantification methods described above: the signal intensity is first quantified for each image pixel of the WSI, then these values are averaged over each zone in each lobulus. To determine zones and lobuli, we used an adaptation of the approach from^21^ illustrated in Fig.  3. Positions of PFs and CVs were detected in the H&E image and transformed to the staining of interest according to the deformation field obtained by image registration. The transformed positions were used to compute lobuli and zones, using the Otsu-based background detection, in the staining of interest.

This proof-of-concept application was chosen to show the feasibility of applying automated PF/CV detection in an otherwise automated zonated quantification. Thus, we deliberately did not choose a biologically relevant question and used very simple and uncalibrated image analysis techniques.

## Results and Discussion

### PF and CV Detection on H&E-Stained Slides

The whole pipeline of training the tile-based Cascade R-CNNs was applied, selecting the one performing best on WSIs (validation data), and evaluating the detection quality on the test data for tiles of different size. The corresponding mean F1 scores are listed in Table 2. Detailed results for the best performing methods can be found in Table 3. The detection results for one of the test cases are illustrated in Supplementary Figure 5. The best performing R-CNNs were consistently found during the first 12 epochs, hence we considered the limit of 24 epochs for training sufficient.

**Table 2:** F1 score averaged over portal field and central vein depending on tile size. Slight, but notable, differences are visible, the maximal F1 score is obtained for a tile size of 1024^2^.

Tile size *s*	512^2^	640^2^	768^2^	896^2^	1024^2^	1280^2^
F1 score	0.782	0.796	0.804	0.807	0.810	0.804

**Table 3:** F1 score, precision and recall for portal field and central vein using a tile size of 1024^2^. Only a slight imbalance between the two classes as well as between precision and recall can be observed.

	TP	FP	FN	Precision	Recall	F1 score
Portal fields	691	196	161	0.779	0.811	0.795
Central veins	600	139	115	0.812	0.839	0.825
Mean	645.5	167.5	138	0.795	0.825	0.810

The highest F1 score of 0.810 was obtained for tiles of size 1024^2^ pixels, corresponding to (930.1 μm)^2^. The differences of the F1 scores between different tile sizes were noticeable, but relatively small. Tile size should be interpreted in combination with resolution and size of the structures of interest: At the resolution used here, a full lobulus or acinus was visible in one tile of size 1024^2^. It is plausible that detection quality does not increase further for tile sizes of 1280^2^ and beyond, as these would not add relevant context, but only increase computational workload and memory requirements. Using smaller tiles, or smaller fields of view at larger resolution, might be useful to reduce memory requirements and increase computational efficiency without substantially compromising detection quality.

Precision and recall, as well as the two classes PF and CV, show only a moderate imbalance (Table 3). Hence, the detector is essentially unbiased with respect to the types of structure, and produces similar numbers of false positives and false negatives.

### PF and CV Detection on Steatotic H&E-Stained Slides

The evaluation of the detection quality on 30 steatotic slides (dataset B) using the previously determined optimal tile size of 1024^2^ lead to the results shown in Table 4.

**Table 4:** F1 score, precision and recall for portal fields and central veins using the detector on a steatotic dataset. Compared to non-steatotic images the detection of portal fields has declined sharply, likely due to the steatosis appearing periportally on this dataset.

	TP	FP	FN	Precision	Recall	F1 score
Portal fields	11061	26299	10936	0.296	0.503	0.373
Central veins	17579	818	7047	0.956	0.714	0.817
Mean	14320	13558.5	8991.5	0.626	0.608	0.595

The detector performs poorer for the steatotic than for the non-steatotic slides. The slides show predominantly periportal steatosis and thus the neighborhood of PFs looks different, so it is to be expected that primarily the detection quality for PFs dropped. Hence, the detector did not generalize well to this type of data not present during training.

### Lobulus Sizes

**Figure 4:**
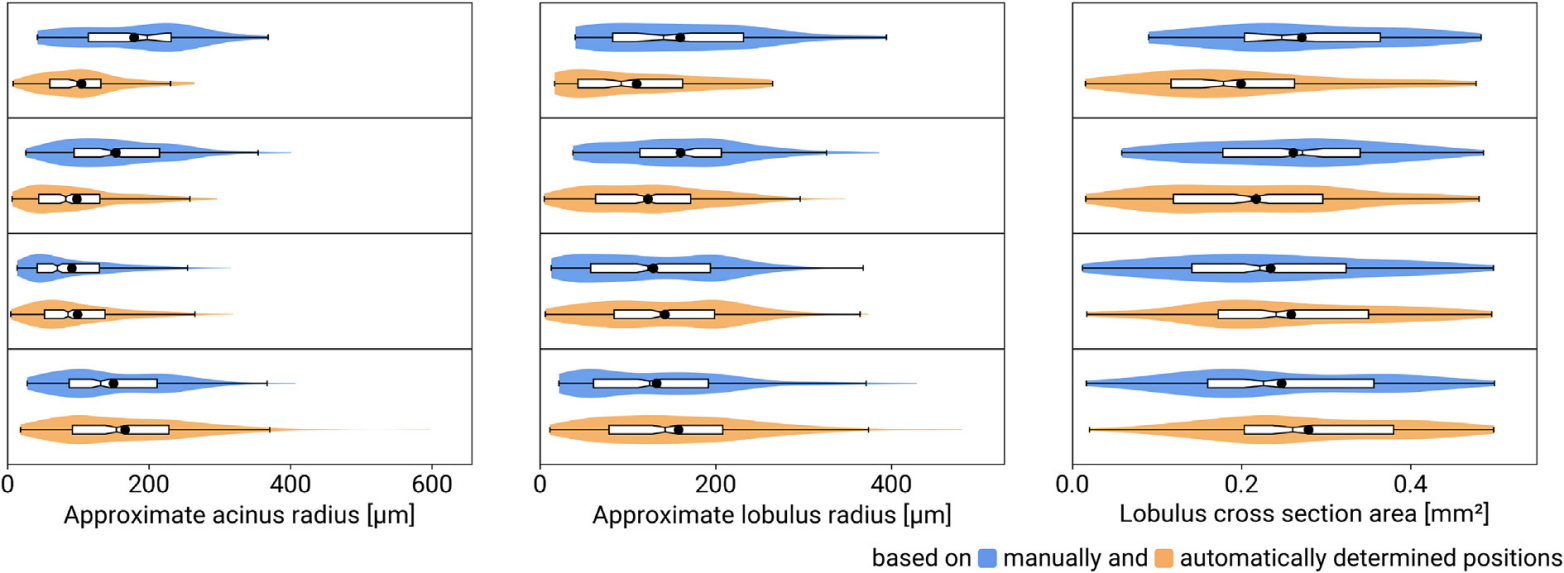
Comparison of approximate acinus radii, lobulus radii, and lobulus cross-section areas (from left to right) for manually (blue, upper plot in each pair) vs. algorithmically (orange, lower plots) obtained points for the four test images. In the box-whisker plots, notches show the 95% confidence intervals of the medians. The violins show the distribution of the values, black dots indicate the respective mean values.

The numbers of PFs, CVs, and lobuli for the test slides of dataset A and manually vs. algorithmically determined points are listed in Table 5. We computed approximate lobulus and acinus radii as well as lobulus areas for the test slides. Results based on the manual annotations vs. results based on the detected PFs and CVs are shown in Fig.  4, computed lobuli for one test image are shown in Supplementary Fig. 6. Comparing the root mean square difference relative to the arithmetic mean, acinus and lobulus radii differ by 36% and 25%, respectively. The corresponding lobulus areas differ by 19%.

**Table 5:** Comparison of the numbers of manual portal field (PF) and central vein (CV) annotations; the numbers of algorithmically determined PF and CV points; and the numbers of lobuli computed from the CV annotations/points for the test images used in the evaluation.

	Manual		Automated		Detected	Lobuli
Test image	# PFs	# CVs	# PFs	# CVs	Manual	Auto
1	45	55	87	91	55	91
2	151	147	268	213	147	210
3	437	288	406	272	287	268
4	219	225	182	192	225	190

The algorithm detected more PF and CV points than there were manual annotations in two of the test images, and fewer for the other two (Table 5). This indicates that the results obtained applying the PF/CV detector are in line with the ones obtained by manual annotation. One image had substantially more PF than CV annotations (437 vs. 288), this difference is reproduced by the algorithm (406 vs. 272 points). The number of computed lobuli compared to the number of CV points shows that almost every CV point is assigned a lobulus. In these images, at most four lobuli per image were in close geometric configurations which the preflooded watershed transform could not handle.

The approximate lobulus cross section areas shown in Fig.  4 generally agreed with previously reported results^9^^,^^21^ of 0.28 mm^2^ and 0.21 mm^2^. The discrepancies between lobulus areas determined from manual annotations vs. algorithmically obtained points corresponded to the discrepancies between approximate radii.

The distributions of approximate radii and areas per WSI corresponded relatively well. This is expected because of the way the geometric properties are computed: The approximate radii are nearest-neighbor distances of the CV positions. The computed lobuli are areas of various shapes depending on the PF and CV positions, where each CV is located close to the geometric midpoint of the corresponding lobulus.

These comparisons between geometric properties computed for the manual annotations and the algorithmically determined points complemented the assessment of detection quality based on the F1 score. The discrepancy between manual annotations and algorithmically determined points had a noticeable impact on the lobulus sizes (Fig.  4). These results are consistent with the previously observed differences in the numbers of detected PFs and CVs (Table 5).

### Zonated Quantification

**Figure 5:**
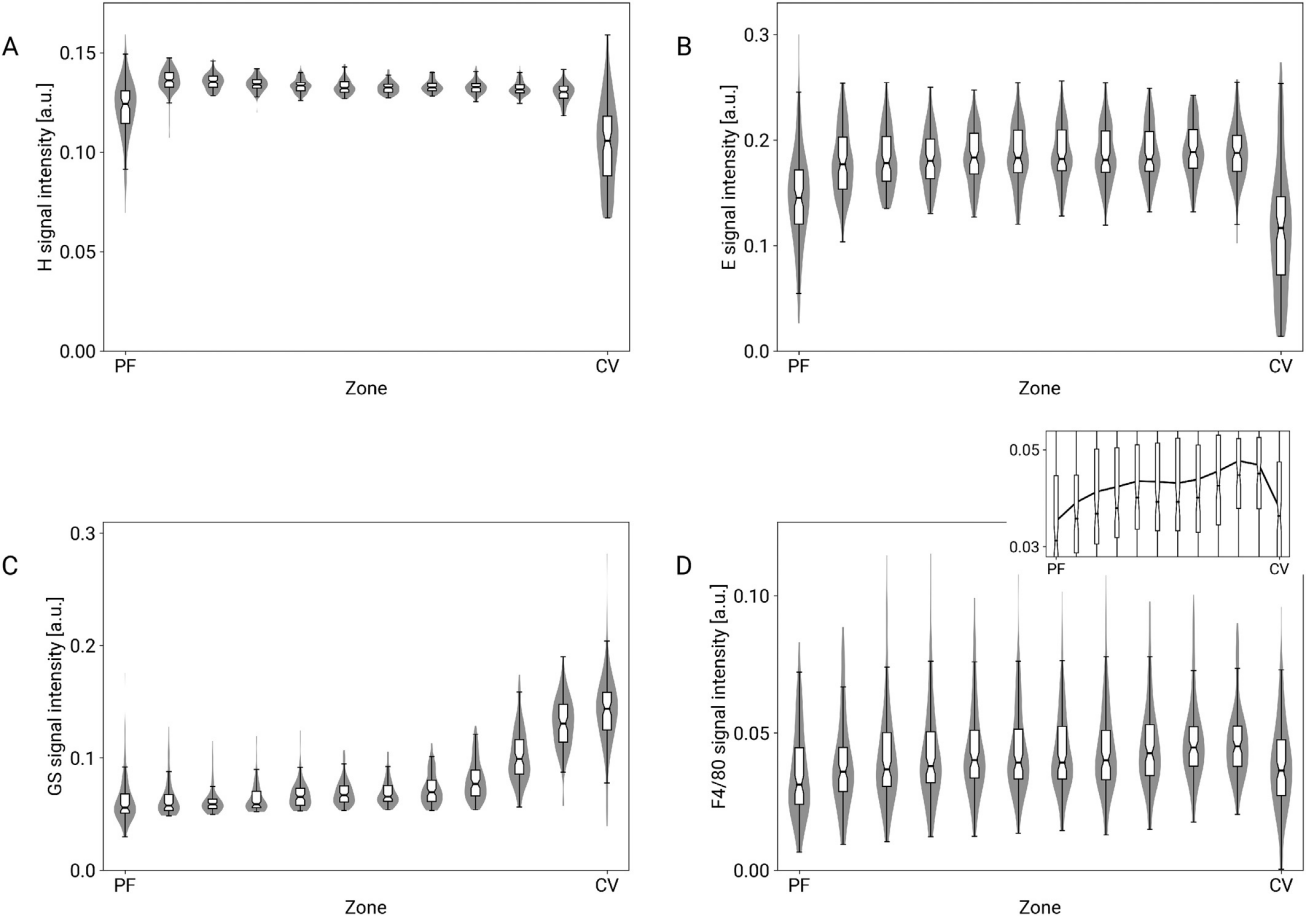
Visualization of signal intensities per zone. Each violin (zone) represents 1/12 of the distance between PF and CV midpoints, i.e., the first and last violin approximately correspond to the vessel structures and violins 1 to 11 correspond to the actual tissue in between. Zonation was not detected in hematoxylin (A) and eosin (B) stained sections, as expected, except for analysis artifacts in the first and last zone. In contrast, the glutamine synthetase signal (GS) was clearly pericentral (C). The F4/80 staining also showed a predominantly pericentral signal (D). In the box-whisker plots, notches show the 95 % confidence intervals of the medians and the violins show the distribution of the values. In the overlayed plot, the black line indicates the respective mean values. The signal detection is not calibrated, so intensities have no absolute interpretation and cannot be compared between plots.

The hematoxylin signal and the eosin signal (negative controls) were homogeneously distributed in all zones, with the exception of the intravascular regions as part of the first and last of twelve zones considered in the analysis (Fig.  5A and 5B). Compared to the hematoxylin signal, the eosin signal was more heterogeneous across the WSI. In contrast, the GS signal (positive control; Fig.  5C) showed a pericentral zonation clearly dominating the artifact of intravascular parts of the first and the last zone. The F4/80 staining heterogeneity (actual proof-of-concept application; Fig.  5D) showed a signal increasing towards the CV. A spatial visualization of the signal intensities per lobulus and zone is shown in Supplementary Fig. 7.

In all these analyses, the first and the last of the 12 zones effectively corresponded to intravascular regions of the WSI. Since the signal of the staining of interest in these regions was close to zero, the corresponding averages over the zone were artificially low. This is a consequence of our current method of computing lobuli and zones based on points for PFs and CVs. This shortcoming could be mitigated in future implementations by segmenting the full PFs and CVs and using their outer contours for computing lobuli and zones. For further discussion of the potential and limitations of the watershed-based lobulus computation from 2D image data and the zonated quantification, we refer to earlier work.^21^

Except for artifacts in the first and last zone, Fig.  5 demonstrated good agreement with our expectations, which were as follows: The negative controls, H&E, were quantified as homogeneous across the zones. The positive control, GS, was quantified as strongly pericentral with substantially lower background or baseline signal of about 0.07 also outside the pericentral region. The F4/80 staining showed a signal with a high baseline and a slight, but still discernible increase towards the CV. This is consistent with the visual impression: the entire tissue appears brown (corresponding to the high baseline) with a more saturated and darker brown pattern predominantly near the CVs in part of the slide (see Supplementary Fig. 4). Even though this staining is biologically nonspecific, it still serves as an example of a detectable difference despite low signal-to-noise ratio.

The number of 12 zones is motivated by the average number of hepatocytes along the radius of lobuli in mice,^9^ and could easily be adapted for other species or for a distinction of fewer zones. Using a fixed number of zones irrespective of the actual lobulus size permits a comparison between lobules depending on the relative location along the PF–CV axis. Depending on the research question at hand, it might be useful to generalize this to a variable number of zones, each corresponding to one layer of hepatocytes.

The signal detection used here as part of the zonated quantification needs to be improved when applying to additional datasets with different characteristics. Instead of a simple color decomposition, one could use a targeted color-to-signal translation, suitable scaling and thresholding of the signal, or entirely different approaches for detecting the tissue regions of interest, such as machine-learning-based approaches. Moreover, intra- and inter-slide heterogeneity of staining needs to be addressed, e.g., by normalization compensating for heterogeneity, or by a color-to-signal conversion analyzing local contrasts in the images. Calibration of the colors/signals is necessary to allow a quantitative interpretation of the analysis results. This could be achieved, e.g., by including in-slide controls.

### Outlook

The automated detection of PFs and CVs yielded good results for the combination of normal and regenerating murine liver tissue (Table 3). The results of a subsequent lobulus size analysis generally agree with literature results (Fig.  4). However, this assessment is based on a dataset of limited size, more annotated images (i.e., more data for calibration and testing) would be needed for a broader evaluation. This additional data should cover a wider range of technical variability, such as tissue prepared in more different laboratories, images acquired by other scanners, and annotations by different observers. Similarly, a generalization of the detector to liver tissue from other species, e.g., humans, will require suitable training data.

The method showed lower detection performance when applied to steatotic tissue (Table 4), a case the algorithm was not trained for. While these data could be included in the calibration of a more general PF/CV detector, this alone would be of very limited benefit, as it would only include a single pathology of single form and etiology from single mouse. The resulting PF/CV detector would likely be biased towards detecting PFs inside steatotic regions, as this is the predominant case present in the data. This problem could possibly be mitigated by suitable data augmentation,^77^ e.g., by creating synthetic lipid vacuoles in different locations or using generative adversarial networks.^86^^,^^87^ Ideally, however, augmentation would be combined with actual images of liver tissue with all pathological alterations to be analyzed.

An extension of the PF/CV detector to stainings other than H&E is conceivable. This would allow a zonated analysis based only on the staining of interest without the need for image registration. Suitable image data covering the relevant variability would be required for training a PF/CV detector for this purpose. The R-CNN used in this study could be used as a starting point for possibly selecting a different CNN architecture or optimizing hyperparameters when generalizing the PF/CV detector. Data augmentation could include virtual stain-to-stain transformations.^88^ We used color normalization (like in the pre-training) for the R-CNN approach here, but color augmentation^89^ might be useful in a generalization.

Computing lobuli based on a single 2D section is also the first step towards determining lobuli in 3D based on serial sections similar to what was done manually in earlier work.^90^^,^^91^ If such a 3D lobulus analysis starts by registering the corresponding WSIs (with potentially different stainings), the detection of PFs and CVs could exploit correlated positions in adjacent slides. Conversely, if a 3D analysis starts by PF and CV detection, image registration could benefit.^92^ Zonated analysis could also be extended to a 3D analysis in registered serial sections, which would require generalizing the geometric analysis (watershed transform and quantization of portality). Moreover, it could be extended to the analysis of further stainings, enabling the 1:1 lobulus-wise correlation of biomarker zonation assessed via different stainings.

The prototypical applications presented here could be extended to investigations of biologically relevant questions. For example, one could compare lobule sizes between healthy, steatotic, and regenerating livers. This will require creating targeted datasets for the specific questions, where the automatic detection of PFs and CVs proposed here reduces the manual image annotation effort. Similarly, comparing zonation of stainings between physiological and pathological states requires targeted datasets for biological research questions, but the annotation effort can again be reduced with the PF/CV detector.

## Conclusions

Our automated PF/CV detector based on a convolutional neural network yields satisfactory results with an F1 score of 0.810 compared to time-consuming manual annotations. However, an F1 score of only 0.595 obtained for a dataset with tissue alterations shows that this approach does not automatically generalize to data unseen in the training. Two proof-of-concept applications illustrate how subsequent automated biomarker quantification in physiologically meaningful regions can build upon automated detection of physiological structures in histological whole-slide images.

## Data Availability

Images and results for datasets A, B, and C as well as the RCNN-based PF/CV detector can be obtained from https://doi.org/10.5281/zenodo.5726769.

## Financial support and sponsorship

This project was funded by the German Federal Ministry of Education and Research (BMBF) via the LiSyM network, grant numbers 031L0040 (DB, LOS, NW), 031L0042 (LAD, IB, UK), 031L0045 (JGH), and 031L0052 (AG). HL was funded by the German Research Foundation (DFG) via the project SteaPKMod (410848700).

## Author contributions

UD created dataset A and dataset B. LAD, IB, UK created the liver samples of dataset C. AG, RH, BBT and JGH stained and scanned the slides of dataset C. NW implemented the tool for manual PF/CV annotation. DB implemented the PF/CV detector and the zonated quantification, performed the analyses. HL implemented the computation of zones and lobuli. LOS implemented the lobulus size analysis. DB and LOS wrote the manuscript. All authors revised the manuscript and approved the final manuscript.

## Conflicts of interest

None.

## Appendix A Supplementary data

Supplementary material Image 1
