# Supplementary material for: Automated Detection of Portal Fields and Central Veins in Whole-Slide Images of Liver Tissue
Source: J Pathol Inform. 2022 Jan 20;13:100001. doi: 10.1016/j.jpi.2022.100001 (PMC8860737; doi:10.1016/j.jpi.2022.100001)
Supplement: Supplementary file 1 — Supplementary material [file mmc1.pdf]

Supporting Figures for
Automated Detection of Portal Fields and Central Veins in Whole-Slide Images of Liver Tissue

Daniel Budelmann[a], Hendrik Laue[b], Nick Weiss[a], Uta Dahmen[c], Lorenza A. D'Alessandro[d], Ina Biermayer[d], Ursula Klingmüller[d], Ahmed Ghallab[e,f], Reham Hassan[e,f], Brigitte Begher-Tibbe[e], Jan G. Hengstler[e], Lars Ole Schwen[b]

[a]*Fraunhofer MEVIS, Lübeck, Germany*
[b]*Fraunhofer MEVIS, Bremen, Germany*
[c]*Experimental Transplantation Surgery, Department of General, Visceral and Vascular Surgery, University Hospital Jena, Jena, Germany*
[d]*Deutsches Krebsforschungszentrum, Systems Biology of Signal Transduction, Heidelberg, Germany*
[e]*Leibniz Research Centre for Working Environment and Human Factors at the Technical University Dortmund, Dortmund, Germany*
[f]*Department of Forensic Medicine and Toxicology, Faculty of Veterinary Medicine, South Valley University, Qena, Egypt*

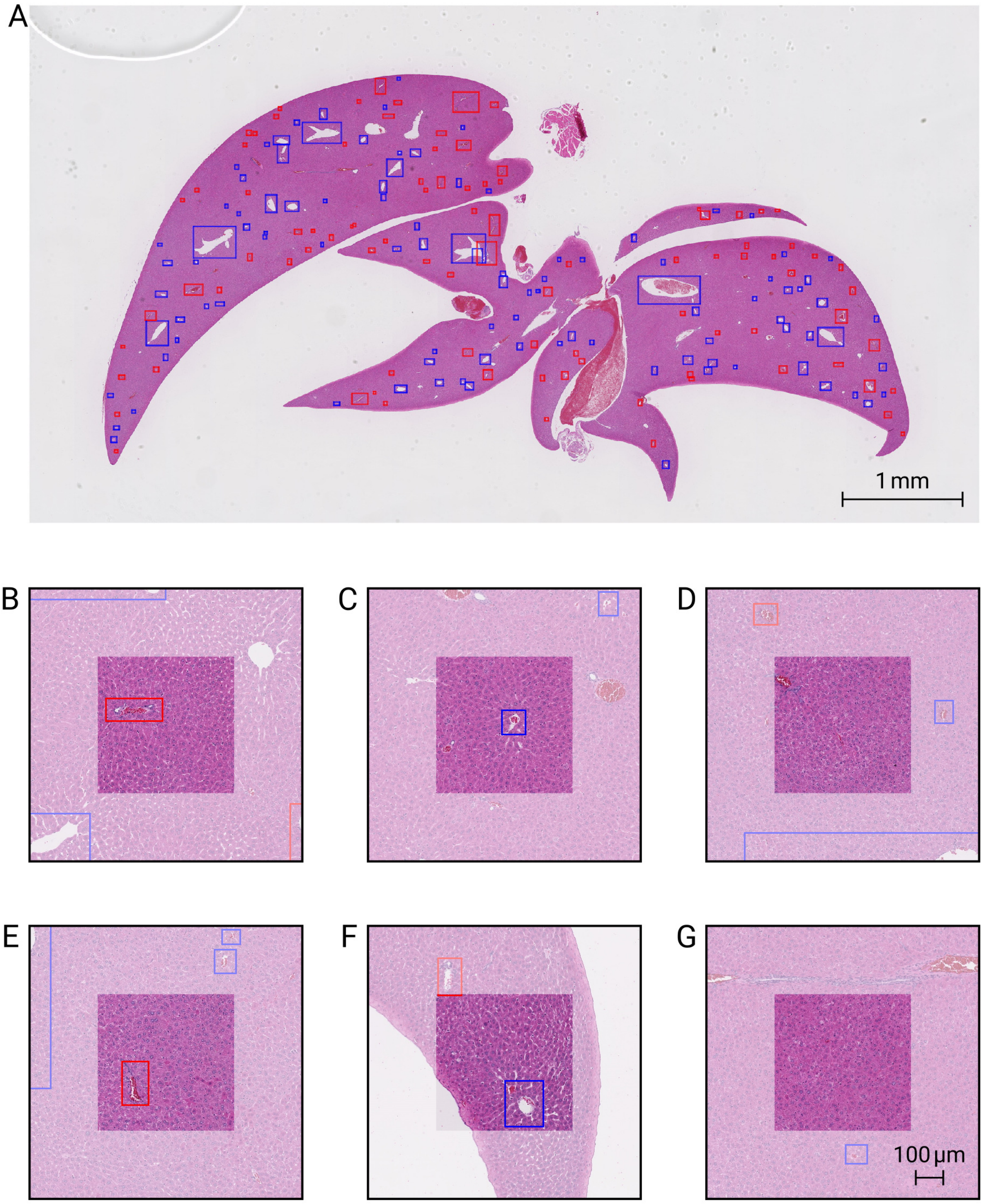

Supporting Figure 1: A: Boxes around portal fields (red) and central veins (blue) were annotated manually (overview image). Below, non-augmented example patches used in the training are shown: two with a portal field in the interior (B and E), two with a central vein (C and F), and two without annotations in the interior (D and G)

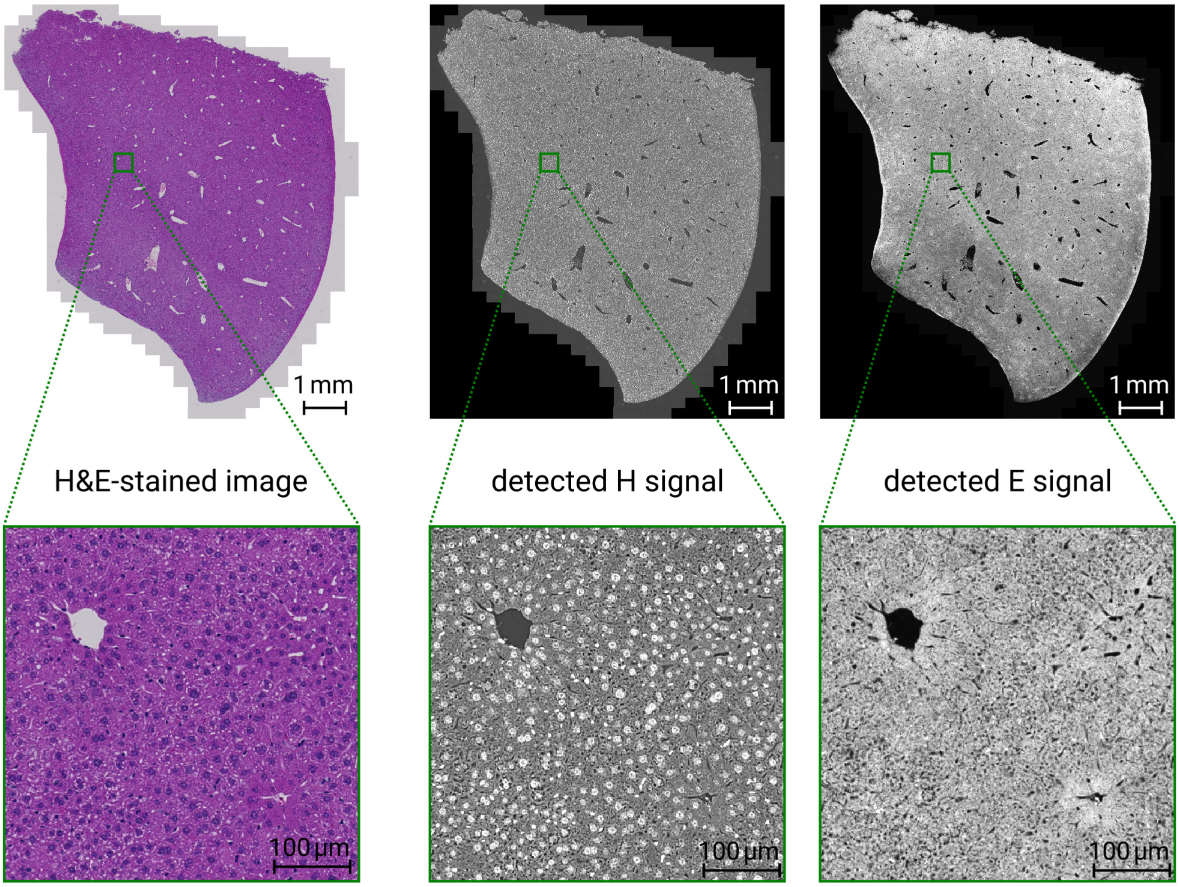

Supporting Figure 2: Decomposition of a whole-slide scan of an H&E-stained (hematoxylin and eosin) histological slide in separate H and E channels.

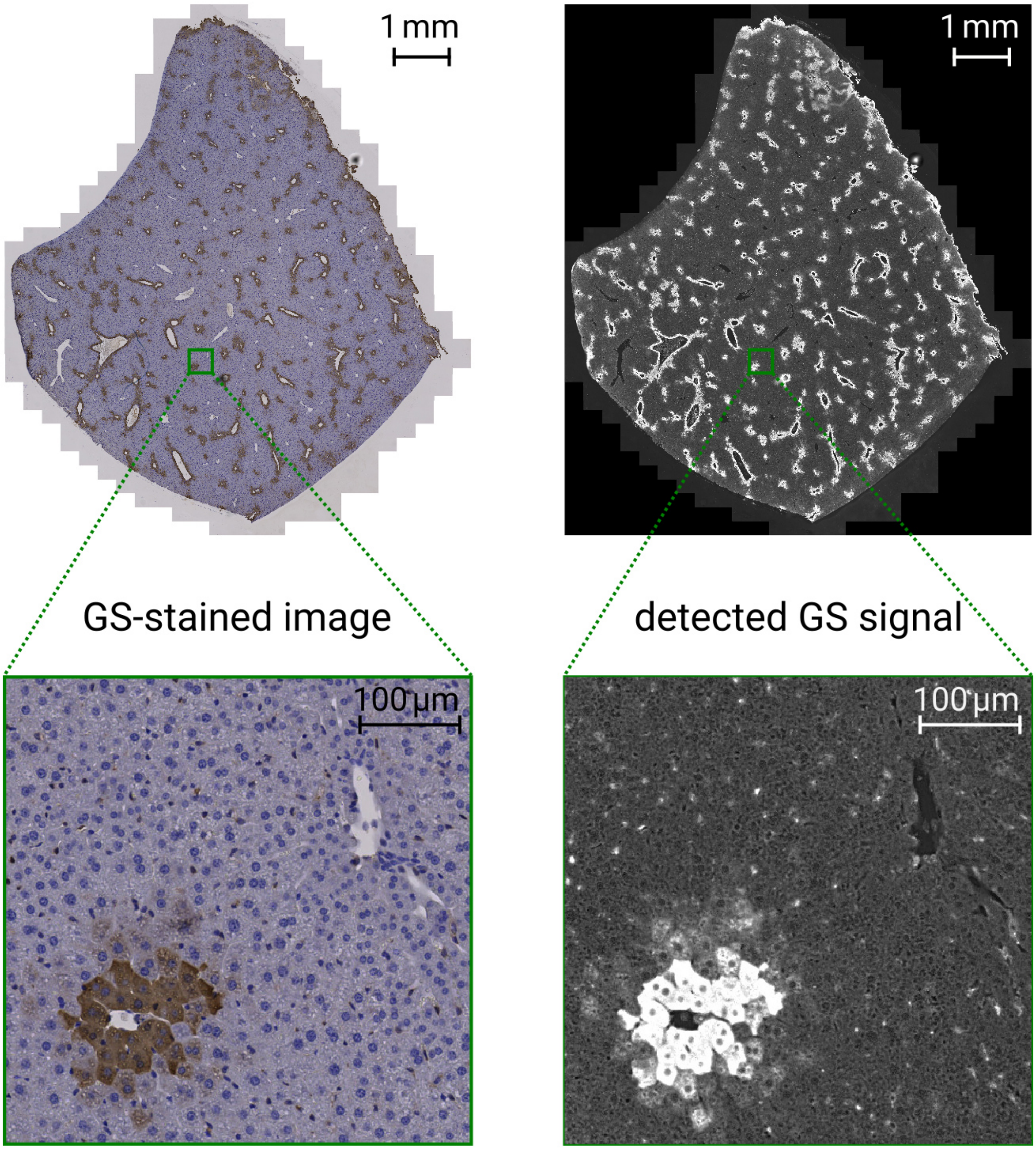

Supporting Figure 3: From a whole-slide scan of a GS-stained (glutamine synthetase) histological slide, a color channel representing the GS signal is extracted.

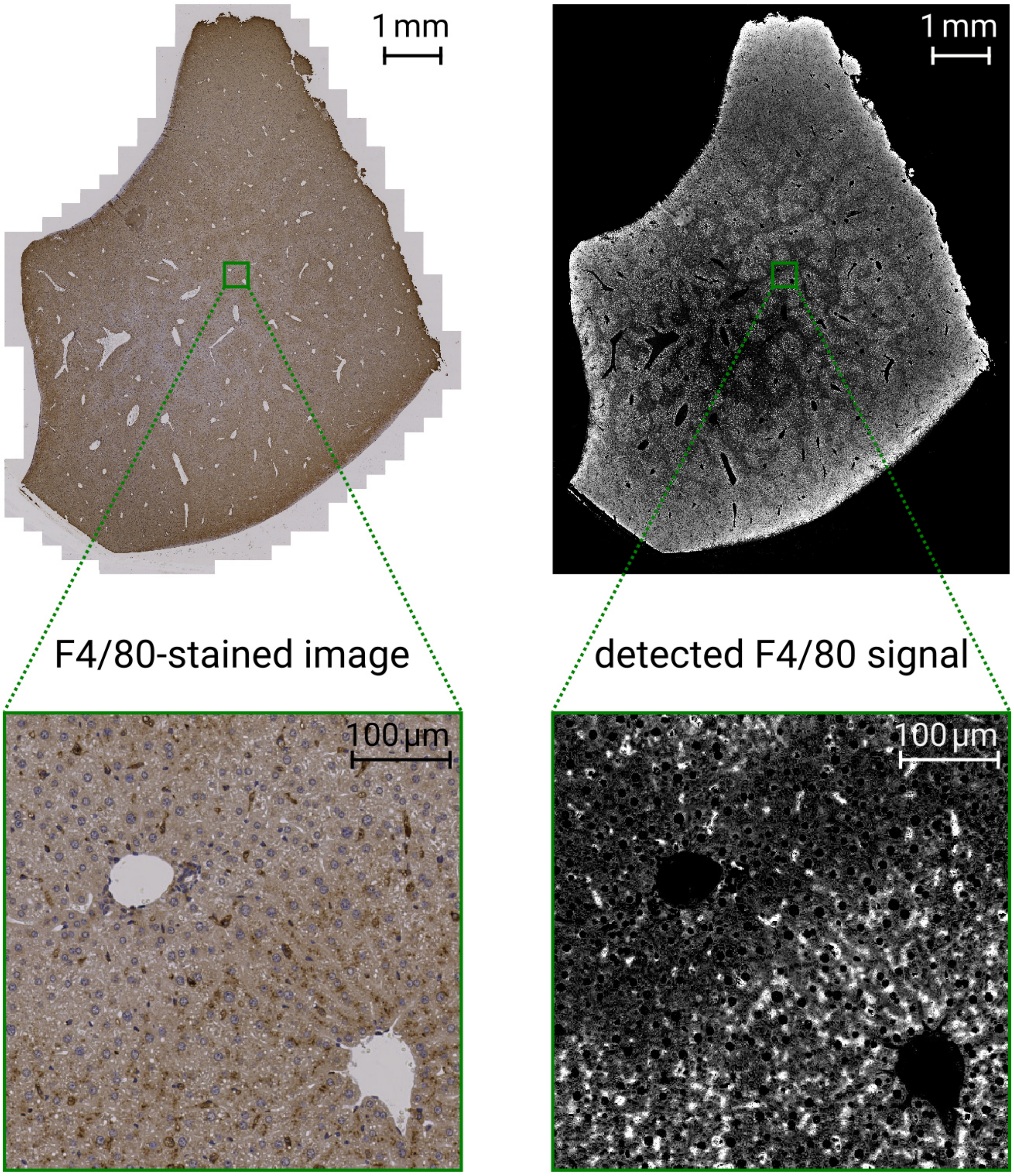

Supporting Figure 4: From a whole-slide scan of an F4/80-stained histological slide, a color channel representing the F4/80 signal is extracted.

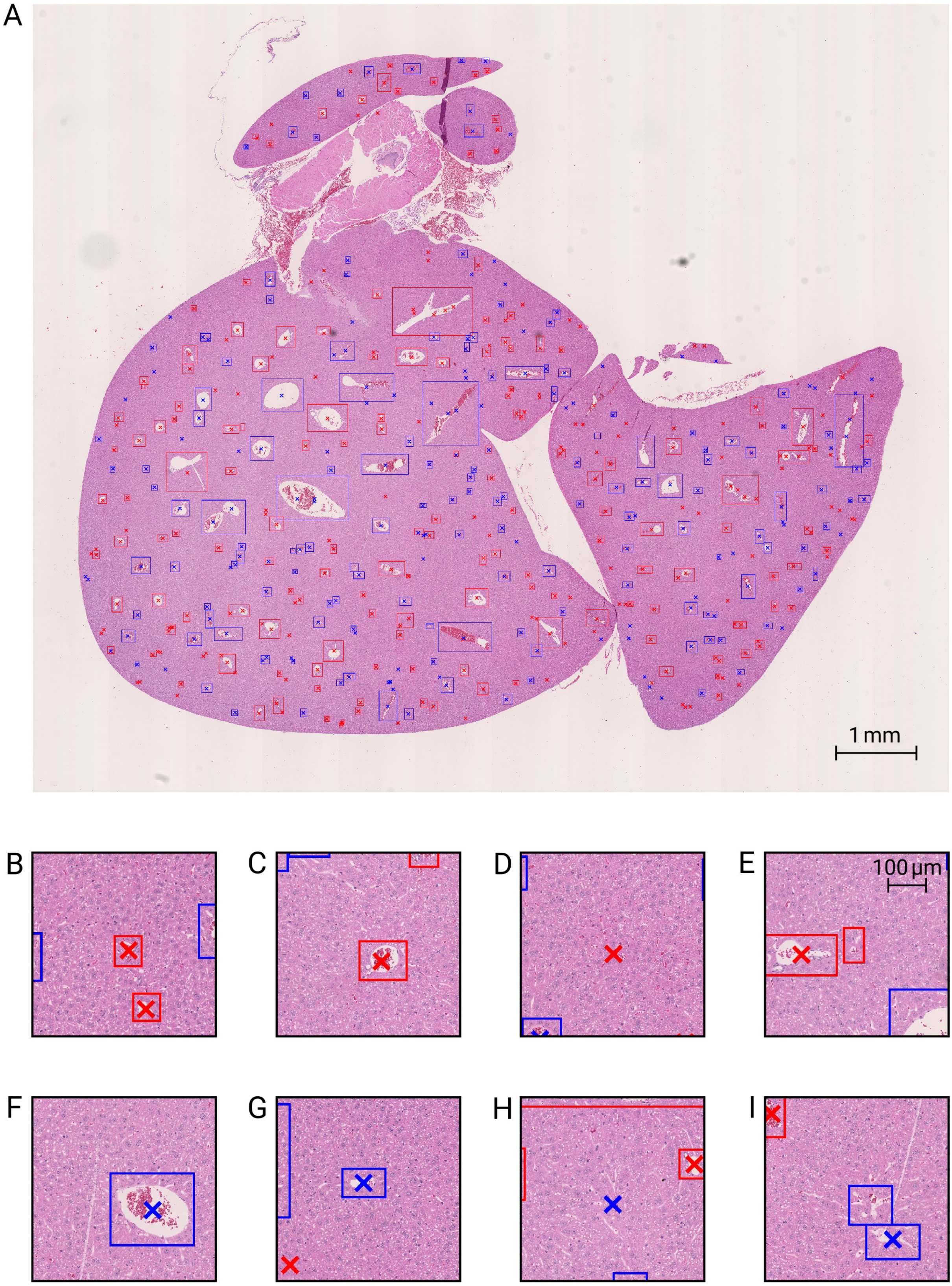

Supporting Figure 5: A: For one test image, algorithmically detected portal field and central vein positions are shown as red and blue crosses, respectively, compared to the corresponding manually annotated boxes (overview image). Below, examples of true positives (B, C, F, and G), false positives (D and H) and false negatives (E and I) are shown.

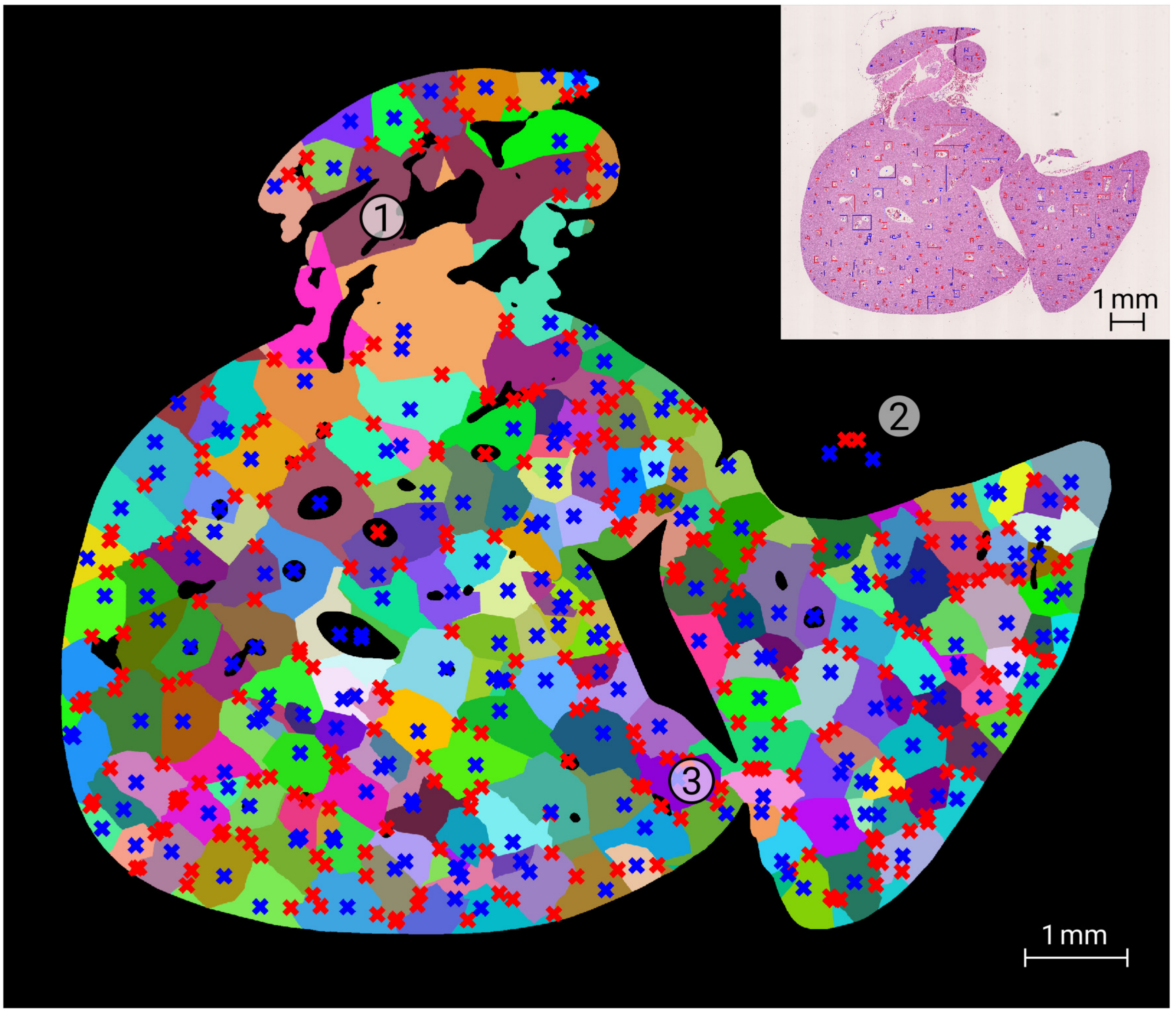

Supporting Figure 6: For the proof-of-concept geometric analysis and zonated quantification, detected portal field (red crosses) and central vein (blue crosses) positions are used to compute lobuli by a watershed transform and by masking background. Computed lobuli for the same dataset as in Supporting Figure 5 are shown in randomized colors. Some minor artifacts can be seen in this example: Non-tissue regions (1) and tissue regions (2), for which the background/tissue detection could be improved; adjacent separate tissue regions (3) which are actually hemodynamically separated, for which lobulus computation based on Euclidian distance produces unphysiological connections.

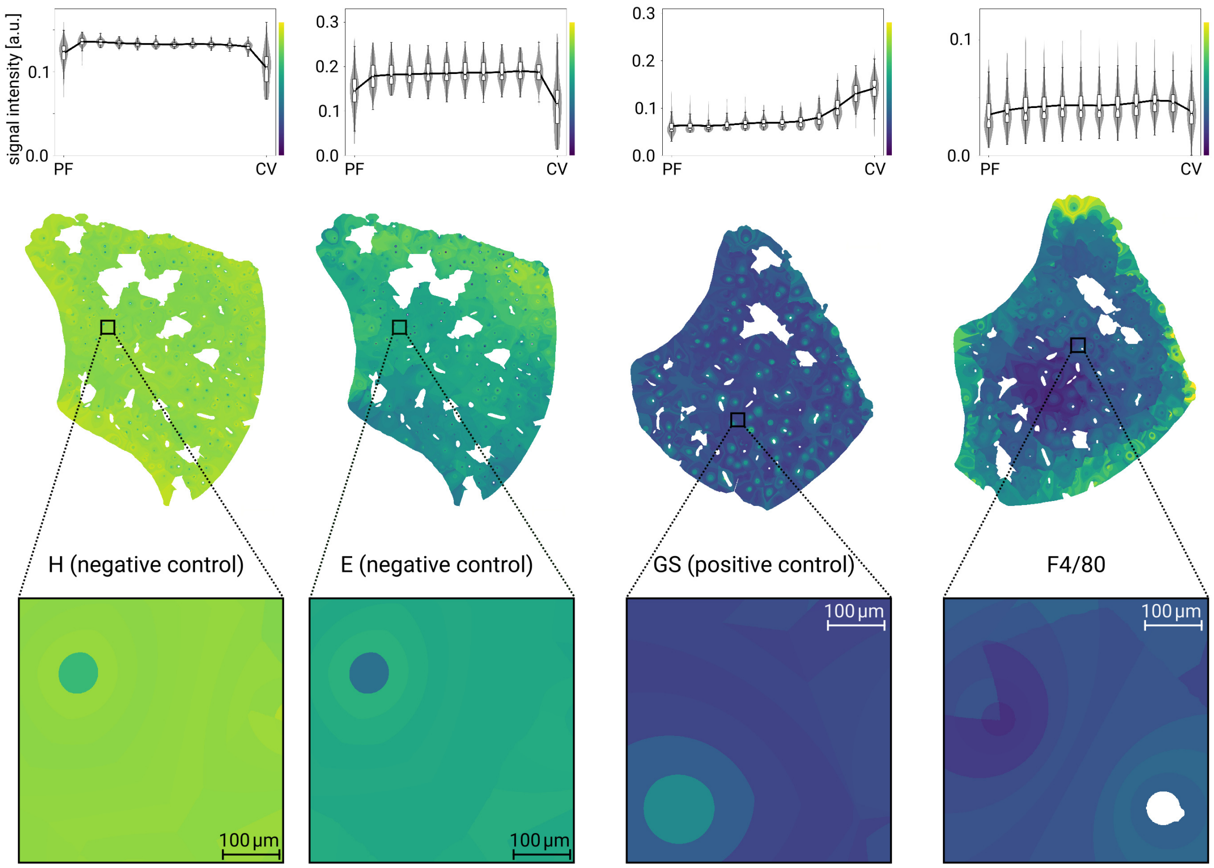

Supporting Figure 7: Visualization of the zonated quantification of the channel signals in the respective lobuli (spatial representation of the data plotted in repeated here in the top row of plots) using a viridis color scale (from dark blue for zero to yellow for maximum signal). Even though the color differences are subtle (matching the value range of the plots in Figure 5), the zonated visualization confirms the trends shown in the plots. The signal detection is not calibrated, so intensities have no absolute interpretation and cannot be compared between plots. The slides and zoomed regions are the same as in Supporting Figures 2, 3, and 4.
